# Precipitation Extremes Under Climate Change

**DOI:** 10.1007/s40641-015-0009-3

**Published:** 2015-04-22

**Authors:** Paul A. O’Gorman

**Affiliations:** Department of Earth, Atmospheric, and Planetary Sciences, Massachusetts Institute of Technology, 77 Massachusetts Avenue, Cambridge, MA 02139 USA

**Keywords:** Extremes, Global warming, Rainfall, Snowfall, Convection, Orographic precipitation, Climate models

## Abstract

The response of precipitation extremes to climate change is considered using results from theory, modeling, and observations, with a focus on the physical factors that control the response. Observations and simulations with climate models show that precipitation extremes intensify in response to a warming climate. However, the sensitivity of precipitation extremes to warming remains uncertain when convection is important, and it may be higher in the tropics than the extratropics. Several physical contributions govern the response of precipitation extremes. The thermodynamic contribution is robust and well understood, but theoretical understanding of the microphysical and dynamical contributions is still being developed. Orographic precipitation extremes and snowfall extremes respond differently from other precipitation extremes and require particular attention. Outstanding research challenges include the influence of mesoscale convective organization, the dependence on the duration considered, and the need to better constrain the sensitivity of tropical precipitation extremes to warming.

## Introduction

The response of precipitation extremes (heavy precipitation events) to climate change has been the subject of extensive study because of the potential impacts on human society and ecosystems [30]. An early study using a four-level general circulation model found that heavy daily precipitation events become more frequent in response to elevated atmospheric CO_2_ concentrations [27]. Numerous model studies since then have also found an intensification of precipitation extremes with climate warming (with important regional variations), and this has been confirmed in the available historical record over land, as will be discussed in detail in later sections.

Understanding of changes in precipitation extremes is better than for changes in other extremes such as tornadoes [44], but large uncertainties and research challenges remain. If changes in dynamics and precipitation efficiency are negligible, precipitation extremes increase with warming because of increases in the saturation vapor pressure of water [4, 62, 85, 86]; this will be made more precise in the “Theory” section. However, dynamical contributions and changes in precipitation efficiency may also play an important role. Mesoscale convective organization is important for the dynamics of precipitation extremes in the tropics (and seasonally in the midlatitudes) but it is not resolved in global models, while at the same time, there are relatively few observational records of tropical precipitation extremes for estimating long-term trends and sensitivities. At higher latitudes, the effect of climate change on snowfall extremes and freezing rain will be different from its effect on rainfall extremes and requires further study. In terms of impacts, the duration of extreme precipitation events and the response of orographic precipitation extremes are both important and are only now receiving substantial research attention.

This paper reviews and elaborates on some of the recent research on how climate change affects precipitation extremes, including observed changes in the historical record (“Observed Changes in Precipitation Extremes”), physical theory (“Theory”), climate-model projections (“Climate-Model Projections”), orographic precipitation extremes (“Orographic Precipitation Extremes”), snowfall extremes (“Snowfall Extremes”), and the duration of precipitation extremes (“Duration of Precipitation Extremes”). The primary focus is on the physical factors that control the intensity of precipitation extremes in different climates. Open questions are discussed throughout and in the “Conclusions and Open Questions” section.

## Observed Changes in Precipitation Extremes

Records of precipitation that are sufficient to detect long-term trends in extremes are primarily from rain gauges over land. Over the available record, there are regions with both increasing and decreasing trends in precipitation extremes [1, 28], as might be expected given large internal variability [25], but the grid boxes or stations with significant increasing trends outnumber those with significant decreasing trends [23, 91•]. Anthropogenic forcing has been shown to have contributed to the intensification of precipitation extremes over northern hemisphere land [53••, 95]. Assessments have also been made of the effect of anthropogenic forcing on the probability of specific extreme precipitation or flooding events using ensembles of climate-model simulations [32, 63, 64•].

**Fig 1:**
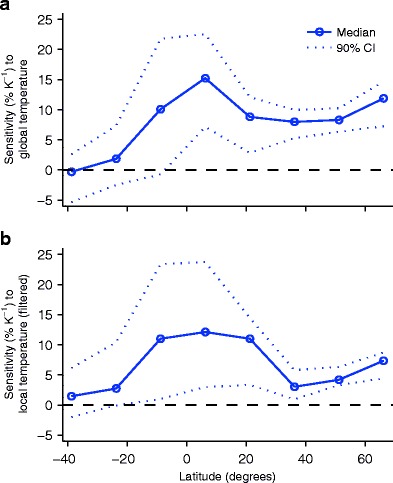
Sensitivities of observed annual-maximum daily precipitation over land (*solid lines with circles*; *dotted lines* show the 90 % confidence interval) in 15° latitude bands relative to **a** global-mean surface temperature or **b** mean surface temperature over the 15° latitude band. Precipitation is from HadEX2, sensitivities are calculated for grid boxes with at least 30 annual values, and the median sensitivity is plotted for each 15° latitude band. Temperatures are over land and ocean from NOAA MLOST, and for **b** the temperature time series were smoothed with a 9-year running-mean filter

One approach that reduces the influence of unforced variability while still distinguishing large-scale variations is to analyze the sensitivity of precipitation extremes averaged over all stations or grid boxes in a latitude band [6, 91•]. Figure 1a shows an example of this type of analysis in which annual-maximum daily precipitation rates over land from the HadEX2-gridded dataset [23] have been regressed over the period 1901 to 2010 against temperature anomalies from NOAA’s Merged Land-Ocean Surface Temperature Analysis (MLOST) [80]. The precipitation rates are over land only, but precipitation extremes do not necessarily scale with the local land mean temperature because of advection of water vapor from over the ocean such as in atmospheric rivers [21, 46], and the temperatures used here are over both land and ocean. For each grid box with at least 30 years of data, the annual-maximum daily precipitation rates are regressed against the global-mean surface temperature anomalies using the Theil-Sen estimator, and the regression coefficient is divided by the mean of the annual-maximum daily precipitation rate at the grid box to give a sensitivity that is expressed in units of percent per kelvin. The median of the sensitivities is then calculated for all grid boxes in 15° latitude bands.^1^ The resulting sensitivity is positive for most latitude bands, the 90 % confidence interval is above zero for all latitude bands in the northern hemisphere, and the global sensitivity (averaging over latitude bands with area weighting) is 8 % K^−1^ with a 90 % confidence interval of 5 to 10 % K^−1^. These results, similar to those obtained previously [6, 91•], provide evidence for an intensification of annual-maximum daily precipitation as the global-mean temperature has risen over the last century and at a rate that is roughly consistent with what might be expected from theory. However, the meridional structure of the sensitivities within the tropics is sensitive to the details of the analysis (cf. [6, 91•]).

Extratropical precipitation extremes at a given latitude occur when the atmosphere is warmer than average and are more closely tied to mean temperatures somewhat further equatorward [21, 61•, 62]. However, they are still expected to respond primarily to changes in mean temperatures in the extratropics rather than the tropics, and recent warming has been greater in the northern extratropics than the tropics. The sensitivities shown in Fig. 1a are based on global-mean surface temperature and do not account for the variation in warming with latitude. Figure 1b shows an alternative analysis in which the annual-maximum daily precipitation rates at each grid box are regressed against the area-weighted mean temperature anomaly for the 15° latitude band that contains the grid box. The latitude-band temperature time series are filtered using a 9-year running mean prior to performing the regression. This filtering reduces the influence of short-term variability in regional temperatures which has previously been found to give a different sensitivity of precipitation extremes than long-term climate change [58]. The results in Fig. 1b show a higher sensitivity of precipitation extremes in the tropics compared to the extratropics, although the uncertainty in the tropics is large reflecting the sparse data there. The sensitivity for the tropics (30S to 30N) is 9 % K^−1^ (90 % confidence interval 6–14 % K^−1^), while for the extratropics, it is 4 % K^−1^ (90 % confidence interval 2–5 % K^−1^). The choice of filter for the temperature time series affects the overall magnitudes of the sensitivities but not whether sensitivities are higher in the tropics than the extratropics. Interestingly, higher sensitivities in the tropics are also found when projections from global climate models are constrained by satellite observations [58] as discussed in the “Tropical Precipitation Extremes” section.

## Theory

To understand the response of precipitation extremes to warming, our starting point is an approximation for the surface precipitation rate in an extreme precipitation event,

1 $$ P\simeq -\varepsilon \left\{\omega (p)S\left(T,p\right)\right\}, $$

where *ε* is a precipitation efficiency, *ω* is the vertical velocity in pressure coordinates (negative for upward motion), $S\left(T,p\right)=d{q}_s/dp\Big|{}_{\theta_e^{*}}$ is the derivative of the saturation specific humidity *q*_*s*_ with respect to pressure *p* taken at constant saturation equivalent potential temperature *θ*_e_^*^ (i.e., the derivative along a moist adiabat), and {⋅} is a mass-weighted vertical integral over the troposphere [56, 61•]. All quantities in Eq. (1) are evaluated locally in the extreme event. The net condensation rate is approximated by − *ωS* either through consideration of the condensation rate in a rising saturated air parcel [62] or using a dry static energy budget in the tropics [56]. The precipitation efficiency *ε* is defined as the ratio of surface precipitation to the column-integrated net condensation; it accounts for condensate and precipitation storage or transport from the column. Note that *ε* is not a conventional precipitation efficiency because it is defined in terms of net condensation (condensation minus evaporation) rather than condensation.

According to Eq. (1), changes in the precipitation rate in extreme events under climate change have a dynamical contribution from changes in *ω*, a thermodynamic contribution from changes in *S* (this is termed thermodynamic since *S* only depends on temperature and pressure), and a microphysical component from changes in the precipitation efficiency *ε*. Relative humidity does not explicitly appear in Eq. (1), but it can affect precipitation extremes through the dynamics and by helping to set the duration of precipitation events. The fractional increase in *S* with warming is influenced by changes in the moist adiabatic lapse rate [11, 62] and varies strongly depending on temperature and therefore altitude in the atmosphere. However, for a moist-adiabatic stratification and convergence confined to near the surface, the thermodynamic contribution can be shown to scale in a similar way to near surface specific humidities [56, 61•, 70]. This scaling is often referred to as Clausius-Clapeyron scaling and gives a sensitivity of 6–7 % K^−1^ for typical surface temperatures. More generally, the thermodynamic contribution depends on the weighting of *S* by the vertical velocity profile in the vertical integral in Eq. (1), and a range of higher and lower rates of change from the thermodynamic contribution have been found in different simulations [56, 61•, 73•]. It is sometimes stated that the dynamical contribution must be positive for a warming climate because of increases in latent heating, but this is not necessarily the case because other factors such as increases in dry static stability or reductions in meridional temperature gradients can counteract the increases in latent heating. Instead, the dynamical contribution is discussed here separately for different dynamical regimes. For example, increases in convective updraft velocities with warming are discussed in the next paragraph, and changes in large-scale vertical velocities in the extratropics are discussed in the “Extratropical Precipitation Extremes” section using the omega equation.

The simplest configuration for which the contributions to changes in precipitation extremes have been analyzed is radiative-convective equilibrium (RCE) in a doubly periodic domain [55•, 56, 70, 77]. There are no large-scale dynamics in RCE, and cloud-system resolving models (CRMs) are used to resolve the convective-scale dynamics. Both the convective available potential energy (CAPE) and the updraft velocities in the middle and upper troposphere increase with warming in RCE [56, 70]; as the atmosphere warms, the thermal stratification remains close to neutral to a strongly entraining plume, and this implies increases in CAPE (calculated based on a non-entraining parcel) and increases in updraft velocities for more weakly entraining plumes [76, 78]. But, the increases in updraft velocities in the upper troposphere do not strongly affect the precipitation extremes, because the factor of *S*(*T, p*) in Eq. (1) gives more weight to the vertical velocities in the lower troposphere in determining the intensity of precipitation extremes. For surface temperatures near those of the present-day tropics, the precipitation extremes increase at close to the thermodynamic rate, and this is close to Clausius-Clapeyron scaling with the surface specific humidity, with relatively small contributions from changes in vertical velocities and precipitation efficiency [56, 70, 77]. The same behavior is found when convection is organized in a squall line [55•]. However, for temperatures below 295 K, the precipitation efficiency can change substantially with warming and the scaling of precipitation extremes then depends on the accumulation period considered [77], as discussed in the “Duration of Precipitation Extremes” section.

## Climate-Model Projections

Climate models provide global coverage for precipitation extremes [39•, 84] and more detailed coverage on regional scales [8•, 22, 36, 37]. They may be applied to different emissions scenarios or individual radiative forcings [18, 35•, 38], and they allow relatively straightforward investigations into the role of dynamics and other factors that contribute to precipitation intensity [24, 61•, 65, 83]. Important limitations in the ability of current models to simulate precipitation extremes have also been recognized and are related in part to the use of parameterized convection [38, 43, 58, 89, 93].

Global models precipitate too frequently with too low a mean precipitation intensity [20, 82], but this does not necessarily mean that they underestimate the intensity of precipitation extremes. For example, in an analysis of 30-year return values of daily precipitation over the conterminous USA, most global climate models were found to overestimate or roughly agree with observations that were conservatively interpolated to the model resolution for comparison [17]. (Appropriate interpolation of precipitation is important because of mismatches in time and space scales between models and observations.) One exception was the Community Climate Model System 3 which underestimated the 30-year return values [17], and increased horizontal resolution [90] or use of superparameterization [49] has been shown to improve the representation of the intensity distribution of precipitation in the Community Atmosphere Model versions 2 and 3. The model bias of too-frequent precipitation mentioned above will affect percentiles calculated over only wet days rather than all days [9], even if the extreme events are properly simulated, which suggests that calculating extremes using all days (or all hours) is preferable for comparison of precipitation extremes between models and observations.

**Fig 2:**
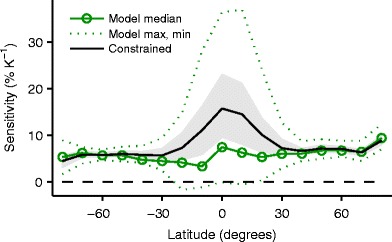
Sensitivity of the 99.9th percentile of daily precipitation to global-mean surface temperature for climate change under the RCP8.5 scenario in CMIP5 global climate-model simulations. Shown are the multimodel median (*green line with circles*) and the full model range (*dotted lines*). Also shown are sensitivities inferred by constraining the model sensitivities using observations of tropical variability (*black line*) with a 90 % confidence interval obtained by bootstrapping as in [58] (*gray shading*)

Projections of twenty-first-century climate change with global climate models show a general increase in the intensity of precipitation extremes except in some regions in the subtropics [38, 39•]. To illustrate basic features of the response, Fig. 2 shows the sensitivity of the 99.9th percentile of daily precipitation to warming as a function of latitude in simulations with 15 global climate models from the Coupled Model Intercomparison Project phase 5 (CMIP5). Sensitivities for climate change (% K^−1^) are calculated as the change in the 99.9th percentile between the final two decades of the twentieth century in the historical simulations and the final two decades of the twenty-first century in the warmer RCP8.5 simulations, normalized by the value in the historical simulations and the change in global-mean surface air temperature.^2^ Note that the sensitivities from observations in Fig. 1 and from simulations in Fig. 2 should not be compared in detail, because of the different time periods, geographic coverage, and measure of extreme precipitation used. We first discuss the simulated response of extratropical precipitation extremes, followed by tropical precipitation extremes and the use of observed variability to better constrain the intermodel spread.

### Extratropical Precipitation Extremes

The multimodel-median sensitivity is shown by the green line with circles in Fig. 2, and the multimodel median of the sensitivity averaged over the extratropics is 6 % K^−1^. A slightly lower extratropical sensitivity of 5 % K^−1^ is obtained if it is normalized by the change in extratropical-mean surface temperature rather than global-mean surface temperature. The intermodel spread in the response (dotted lines in Fig. 2) is small in the extratropics, consistent with the fact that most extratropical precipitation extremes are associated with cyclones and fronts [13•, 69] that may be expected to be reasonably well simulated. However, global models with conventional parameterizations are unable to simulate precipitation extremes from mesoscale convective systems over midlatitudes in summer [43], and so the results from these models are not reliable for regions and times of year in which these systems are important.

Equation (1) with the precipitation efficiency *ε* held fixed reproduces the fractional changes in precipitation extremes in CMIP3 simulations [61•, 83]. The thermodynamic contribution in these simulations is close to what would be expected from scaling of precipitation extremes with surface specific humidity, and this implies a lower rate of increase than scaling with column water vapor [60]. In the extratropics, the simulated rate of increase of precipitation extremes is close to the thermodynamic contribution at all latitudes, and there is little dynamical contribution from changes in vertical velocities [24, 61•]. A stronger dynamical contribution resembling a poleward shift has been found in idealized aquaplanet simulations [51, 62].

Why is there no general strengthening or weakening of large-scale vertical velocities associated with simulated extratropical precipitation extremes despite changes in latent heating and dry static stability? As a starting point, consider the quasigeostrophic omega equation written as

2 $$ {\nabla}^2\left(\sigma \omega +\frac{\kappa J}{p}\right)+{f}_0^2\frac{\partial^2}{\partial {p}^2}\omega =\mathrm{R}\mathrm{H}\mathrm{S}, $$

where *ω* is the vertical velocity in pressure coordinates, *σ* is the dry static stability parameter, *J* is the diabatic heating rate, *κ* is the ratio of the gas constant to the specific heat capacity at constant pressure, *p* is pressure, *f*_0_ is a reference value of the Coriolis parameter, and the right-hand-side (RHS) includes vorticity and temperature advection terms but not the static stability or diabatic heating rate [34]. This equation is the simplest equation for the vertical velocity that accounts for dynamical balance, and it is used here to gain some insight into the controls on large-scale vertical velocities in the extratropics, although it is not expected to be quantitatively accurate. In a strong non-convective event with saturated ascent, *J* will be dominated by latent heating and *σ* + *κJ*/(*pω*) is a measure of the moist static stability. This moist static stability will be small if the stratification is close to moist adiabatic, as was the case, for example, in the extreme precipitation event in the Colorado Front Range in September 2013 [26]. For a region of upward motion that is sufficiently broad in the horizontal with small moist static stability, Eq. (2) reduces to ${f}_0^2\frac{\partial^2}{\partial {p}^2}\omega \simeq \mathrm{R}\mathrm{H}\mathrm{S}$, and the effect of climate change on the vertical velocity *ω* does not depend on changes in static stability or latent heating. The vertical velocity still depends on RHS, but changes in this would be expected to be relatively small given modest changes in eddy kinetic energy [57] and eddy length [40].

Equation (2) gives, therefore, some insight as to why the vertical velocities associated with large-scale extratropical precipitation extremes might not change greatly under climate change. The term proportional to *f*_0_^2^ on the left hand side of Eq. (2) arises from planetary rotation, and it makes the large-scale vertical velocity much less sensitive to deviations from a moist adiabatic stratification when compared to small-scale convective updrafts (see the “Theory” and “Duration of Precipitation Extremes” sections). We next turn to the tropics where the dynamical influence of planetary rotation is weaker and where convection is always a key factor for precipitation extremes.

### Tropical Precipitation Extremes

As compared to the extratropics, the intermodel range in the sensitivity of precipitation extremes is much larger in the tropics (Fig. 2), with close to zero sensitivity in some models and greater than 30 % K^−1^ in others. Additional reasons to doubt the response of tropical precipitation extremes in these global climate models include the large differences between tropical precipitation extremes in the twentieth-century simulations in different models [38], the inability of the models to represent mesoscale convective organization [72] or to simulate the interannual variability in tropical precipitation extremes when compared to observations [2, 3], and the disproportionate increases in precipitation extremes compared to other parts of the precipitation distribution that is found in some models—an “extreme mode” in the tropical response to climate change that relates to gridpoint storms [66, 67].

Observations can be used to better constrain the large uncertainty in the response of tropical precipitation extremes to warming. The sensitivity of tropical precipitation extremes for climate change in different climate models is correlated with their sensitivity for shorter term variability within a climate (variability that is primarily related to El Niño-Southern Oscillation) [58]. For example, models with a relatively high sensitivity of tropical precipitation extremes for climate change also have a relatively high sensitivity of tropical precipitation extremes for variability in historical simulations, although the sensitivities for climate change and variability are generally different in value. The robust relationship between the sensitivities for climate change and variability has been used together with observed variability to constrain the sensitivity of tropical precipitation extremes to climate change [58]. The black line in Fig. 2 shows a similar observationally constrained estimate of the sensitivity of the 99.9th percentile of daily precipitation for climate change, but instead of considering the sensitivity for climate change aggregated over the whole tropics as in [58], the analysis is applied separately to the sensitivity for climate change in 10° latitude bands in both the tropics and extratropics.^3^ This observationally constrained estimate is similar to the multimodel median in the extratropics but higher than the multimodel median in the tropics. It peaks near the equator and is higher for the tropics (11 % K^−1^, 90 % confidence interval 7–15 % K^−1^) than the extratropics (6 % K^−1^, 90 % confidence interval 6–7 % K^−1^). Interestingly, a higher sensitivity in the tropics compared to the extratropics was also found using historical rain-gauge data (“Observed Changes in Precipitation Extremes”). For the tropics, there still remains considerable uncertainty in both the estimate from rain-gauge data and the observationally constrained estimate discussed in this section, and better constraining the sensitivity of tropical precipitation extremes is an important research challenge.

## Orographic Precipitation Extremes

Idealized simulations have recently been used to study the response of orographic precipitation extremes to climate warming [73•, 74•] (see also [41] for a more general discussion). A striking result from these studies is that there are higher fractional changes in precipitation extremes on the climatological leeward slope of the mountain as compared to the windward slope. Orographic precipitation extremes must be treated as a special case for several reasons. The thermodynamic contribution is influenced by the vertical profile of the vertical velocity (see Eq. 1), and the shape of this profile will generally be different over a sloped lower boundary than over a flat lower boundary [74•]. Downstream transport of precipitation means that the local precipitation efficiency can vary strongly over the mountain, and the condensation that leads to leeward precipitation may occur relatively high in the atmosphere where sensitivities to temperature change are greater [74•]. In addition, changes in vertical velocities are governed by mountain wave dynamics and have been found to be different for extreme precipitation events on the western and eastern slopes of an idealized midlatitude mountain [73•].

A weakening of orographic rain shadows related to changes in precipitation extremes has previously been noted in simulations of climate warming over North America [22, 75]. Further study is needed to assess the role played by the physical factors discussed above in determining changes in orographic precipitation extremes in comprehensive simulations and observations.

## Snowfall Extremes

Changes in snowfall extremes have received relatively little research attention, party because of the difficulties in producing long-term records of snowfall. Observational studies of daily snowfall extremes have been regional in nature and have found large interdecadal variability with, for example, no long-term trend for Canada [94] but more frequent extreme snowstorms in recent decades in the eastern two thirds of the USA [44]. Studies using different metrics have reached different conclusions as to whether there are more heavy snowfall events in anomalously warm or cold years or seasons in the USA [15, 44].

Physically, snowfall extremes are expected to be affected by climate warming through both increases in saturation vapor pressures and changes in the frequency of occurrence of temperatures below the rain-snow transition temperature. A simple asymptotic theory of snowfall extremes has been developed based on the temperature dependencies of precipitation extremes and the rain-snow transition [59•]. According to the simple theory, snowfall extremes tend to occur near an optimal temperature of roughly −2 °C when snowfall is measured by liquid water equivalent. The optimal temperature arises because saturation vapor pressures increase with temperature whereas the fraction of precipitation that falls as snow reduces sharply at surface temperatures near freezing. When snowfall is measured by depth of snow, the optimal temperature is lower (roughly −4 °C) because the variation of snow density with temperature must also be taken into account. For an infinitesimal climate warming, the intensity of snowfall extremes decreases for climatological-mean temperatures above the optimal temperature and increases for climatological-mean temperatures below it. Furthermore, fractional changes in high percentiles of snowfall are smaller the higher the percentile considered (unlike for rainfall extremes), and fractional changes in the intensity of the most extreme events tend to be relatively small. There may still be large fractional decreases in snowfall extremes with warming in regions with climatologically mild temperatures, and changes in the frequency of exceeding a fixed high threshold of snowfall may still be substantial.

Snowfall extremes in simulations with global climate models from CMIP5 behave similarly to the simple theory for sufficiently extreme statistics [59•], although the climatological temperature below which snowfall extremes intensify is lower than the simple theory predicts. The response of snowfall extremes is similar in the subset of models that most realistically simulate Arctic sea ice [59•], the decline of which has been hypothesized to affect midlatitude weather extremes [19].

Regional climate-model simulations exhibit large fractional decreases in maximum winter daily snowfall over much of western Europe, but little change or increases in other parts of Europe that are climatologically colder [88]. As in the simple theory and in global climate-model simulations, there is a strong link in regional simulations [88] and downscaled global simulations [52] between the changes in snowfall extremes and the local climatological temperature in the control climate.

## Duration of Precipitation Extremes

The impact of changes in precipitation extremes depends on the duration of precipitation considered (i.e., the accumulation period). In a recent climate-model study, intensity-duration-frequency curves were calculated for accumulation periods from 6 h to 10 days, and the curves were found to shift upwards in intensity on a logarithmic scale in a relatively simple way as the climate warms [35•]. However, it is not clear that global climate models can be relied on for subdaily extremes, because of the potential importance of convective processes. Indeed, for regional simulations of midlatitudes in summer, changing from a model with convective parameterization to a CRM has been found to lead to a marked improvement in the intensity distribution of hourly precipitation [8•] and to significantly alter the simulated response of hourly precipitation extremes to climate change [37].

Long-term observational records of subdaily precipitation are relatively sparse, which makes it difficult to give a general assessment of trends in subdaily extremes [92]. Many recent observational studies have instead focussed on the relationship between short-duration precipitation rates and the local surface temperature in variability within the current climate. In the first of these studies, a high-resolution record from the Netherlands was found to give a sensitivity of 7 % K^−1^ for daily precipitation as compared to 14 % K^−1^ for hourly precipitation over a range of temperatures [47]. Similar behavior was found in some but not all subsequent studies in different regions [10, 31, 48, 54, 87]; see [92] for an in-depth discussion. Factors such as relative humidity [31, 48], large-scale dynamics and temperature gradients [54], and transitions from stratiform to convective precipitation [10, 29] are thought to be important for the scaling of precipitation extremes with temperature in the current climate, and some of these factors may have a different effect on hourly and daily precipitation. While the sensitivity of precipitation extremes for long-term climate change need not be the same as for variability in a given climate [9, 58], understanding the sensitivity of subdaily precipitation extremes in the present-day climate is an important starting point.

Idealized CRM studies suggest that changes in both dynamics and precipitation efficiency could contribute to the scaling of subdaily convective precipitation extremes with temperature. Convective precipitation extremes have been found to increase with warming considerably faster than implied by Clausius-Clapeyron scaling in some cases when a temperature increase is imposed that is constant in the vertical [7, 79]. This is not surprising because a vertically uniform temperature increase makes a moist atmosphere less statically stable and leads to faster updrafts [50•], but it does demonstrate that changes in the static stability associated with subdaily extreme precipitation events are worthy of further study. In a related result, temperature changes in climate-change simulations were found to be close to constant in the vertical for high-CAPE composites in the midlatitudes [7, 50•].

**Fig 3:**
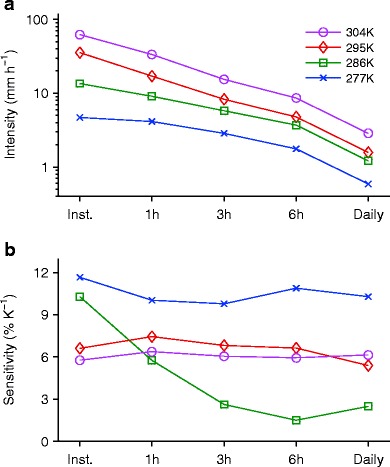
**a** The 99.99th percentile of precipitation for different durations (instantaneous, 1 h, 3 h, 6 h, and daily) in simulations of radiative-convective equilibrium with a cloud-system resolving model at selected mean surface-air temperatures as given in the legend. **b** The sensitivity of the 99.99th percentile of precipitation to mean surface air temperature changes for the same temperatures shown in (**a**). The natural logarithm of the 99.99th percentile of precipitation as a function of mean surface-air temperature from ten simulations is linearly interpolated to a uniform grid in temperature and sensitivities (% K^−1^) are calculated as the change for a 3-K warming

As discussed in the “Theory” section, updrafts do become somewhat faster with warming when lapse rates are allowed to equilibrate (rather than being imposed) in simulations of RCE, although the dynamical contribution to changes in precipitation extremes is still relatively small [56, 70, 77]. Nonetheless, large deviations from Clausius-Clapeyron scaling have been found in a study of RCE because of changes in precipitation efficiency at mean surface temperatures below 295 K [77]. The 99.99th percentile of precipitation from this study is shown in Fig. 3a for durations from instantaneous to daily.^4^ Warming shifts the percentile curves upwards in intensity in Fig. 3a, but the rate at which they shift upwards varies with duration and temperature. As shown in Fig. 3b, the precipitation extremes follow Clausius-Clapeyron scaling at roughly 6–7 % K^−1^ for temperatures above 295 K. However, for temperatures below 295 K, the sensitivity varies widely depending on temperature and accumulation period in a manner that is not fully understood. Instantaneous precipitation extremes increase at close to double the Clausius-Clapeyron rate for temperatures below 295 K, and this has been shown to be due to increases in precipitation efficiency with warming, related in part to increases in hydrometeor fall speed as more of the precipitation in the column changes from solid to liquid [77]. Such changes in precipitation efficiency might be expected to occur for variability within a climate as well as for longer term climate change, but in the simulations, they depend strongly on the choice of cloud microphysics scheme, and it remains to be seen if they are relevant for observed precipitation extremes.

## Conclusions and Open Questions

As demonstrated in several observational studies, there has been an overall intensification of daily precipitation extremes as a result of global warming, although the available data has limited geographic coverage, and there are large regional variations in the observed trends. Much of the characterization of projected changes in precipitation extremes comes from climate models that use parameterized moist convection, but these are not expected to be reliable for precipitation extremes that are primarily convective in nature (for example, in the tropics or for certain events in summer in the extratropics). As a result, simulations that use cloud-system resolving models or superparameterizations are becoming increasingly important to research in this area. Even when convective dynamics are resolved, precipitation extremes at short durations have been found to be sensitive to the parameterization of cloud and precipitation microphysics [77], and progress in observations and physical understanding remains equally important.

Contributions from changes in thermodynamics, dynamics, and precipitation efficiency have all been found to be important for changes in precipitation extremes in at least some situations in modeling studies. The thermodynamic contribution is the easiest to understand and always gives an intensification with warming. There is some basic understanding of dynamical contributions at the large scale from the omega equation (“Extratropical Precipitation Extremes”) and also at the convective scale in the case of RCE (“Theory” and “Duration of Precipitation Extremes”), but only a few studies have focussed on the role of mesoscale convective organization in precipitation extremes [55•, 72, 79].

Precipitation extremes associated with particular dynamical regimes or particular precipitation types may respond differently to climate warming and are deserving of special attention. As discussed in the “Orographic Precipitation Extremes” section, recent idealized studies of orographic precipitation extremes have found that fractional increases are larger on the climatological leeward side than on the windward side, and further work is needed to relate this to more realistic modeling studies and observations. Similarly, snowfall extremes behave quite differently from rainfall extremes because they tend to occur near an optimal temperature that is unaffected by climate warming. Further work is needed to understand the specific responses of lake-effect and high-elevation snowfall extremes, as well as changes in the frequency of hail and ice storms [14, 16].

Characterizing the dependence of changes in precipitation extremes on duration is of importance for impacts, and this is particularly challenging for subdaily durations. Much research has focussed on precipitation accumulated over fixed time periods as discussed in the “Duration of Precipitation Extremes” section. An alternative approach is to consider properties of contiguous precipitation events that are defined based on when non-zero precipitation begins and ends [10, 68, 71]. Consideration of the amount of precipitation in a given event (the event depth) may be advantageous because observed distributions of event depths exhibit a power law range [68, 81] and thus, their response to climate change may be relatively simple to characterize.

Daily precipitation extremes in the tropics seem to be more sensitive to climate warming than those in the extratropics, as suggested by results from both rain-gauge observations (“Observed Changes in Precipitation Extremes”) and climate-model projections constrained using satellite observations (“Tropical Precipitation Extremes”). One possible cause is a more positive dynamical contribution in the tropics than the extratropics. Changes in extratropical eddy kinetic energy are relatively modest and can be either positive or negative depending on the season and hemisphere [57], whereas increases in the frequency of the most intense tropical cyclones are expected as the climate warms [42], and tropical cyclones contribute substantially to off-equatorial precipitation extremes in the current climate [45]. Furthermore, increases in the activity of the Madden-Julian Oscillation and convectively coupled equatorial Kelvin waves have been found in simulations with conventional and superparameterized climate models [5, 12]. The influence of these potential dynamical changes on the aggregate statistics of tropical precipitation extremes remains to be assessed.

## Footnotes

1 The circles in Fig. 1 are plotted at the midpoints of the latitude bands. There are relatively few grid boxes for some latitude bands, and higher-latitude bands with little data are excluded. Uncertainty is estimated by bootstrapping the years used at each grid box (1000 bootstrap samples are generated) and then calculating a 90 % confidence interval for the median sensitivity in each latitude band (or averaged over several latitude bands).

2 The model names and the exact time periods used are given in [58]. The daily precipitation rates are first conservatively interpolated [17] to an equal-area grid with constant spacing in longitude of 3°. Following [61], the precipitation extremes in a given climate are calculated by aggregating daily precipitation rates (over both land and ocean and including dry days) at a given latitude and then calculating the 99.9th percentile. Calculating the change in precipitation extremes at each grid box and then taking the zonal average has been found to give similar results [65]. The sensitivities are averaged over 10° latitude bands for presentation in Fig. 2.

3 The observationally constrained estimate is obtained by regressing the sensitivity for climate change against the sensitivity for variability across the models, and then using this regression relationship together with the observed sensitivity for variability to estimate the sensitivity for climate change. The sensitivity for variability (% K^−1^) is calculated in both models and observations based on the 99.9th percentile of daily precipitation rates aggregated over the tropical oceans (30S to 30N) and the mean surface temperature over the tropical oceans, as described in detail in [58]. The sensitivities for climate change are calculated in 10° latitude bands relative to the change in global-mean surface temperature, as for the other sensitivities shown in Fig. 2. Differences in convective parameterizations are less important in the extratropics, and the correlation coefficient across models between the sensitivities for climate change and variability becomes smaller for climate change at higher latitudes, reaching a value of 0.5 at 50S and 40N as compared to a maximum of 0.86 at 20N. SSM/I data from Remote Sensing Systems [33] are used for the observed precipitation rates and NOAA MLOST data [80] for the observed temperatures.

4 Percentiles are calculated including zero precipitation amounts, and the simulations have 500-m horizontal grid spacing and use the cloud microphysics scheme referred to as “Lin-hail” in [77].
